# Trichobezoar-associated jejunojejunal intussusception: an exceptionally rare manifestation of Rapunzel syndrome

**DOI:** 10.1093/jscr/rjag258

**Published:** 2026-04-15

**Authors:** Shashwat Vyas, Hemant Sharma

**Affiliations:** Department of General Surgery, Dr SS Tantia Medical College, Hospital & Research Centre, Hanumangarh Road, Sriganganagar, Rajasthan, 335002, India; Department of Renal Transplant Surgery, Royal Liverpool University Hospital, Liverpool University Hospitals NHS Foundation Trust, Prescot Street, Liverpool, L7 8XP United Kingdom

**Keywords:** Rapunzel syndrome, rare variant, trichobezoar, intussusception

## Abstract

Rapunzel syndrome, a rare type of trichobezoar where a hairball in the stomach extends into the small intestine through the pylorus. Intussusception associated with Rapunzel syndrome is particularly rare, with fewer than 10 reported cases worldwide. This report presents a 20-year-old woman with jejunojejunal intussusception caused by Rapunzel syndrome. She had 3 months of upper abdominal pain, vomiting, early fullness, weakness, and noticeable abdominal mass. Contrast-enhanced computed tomography scan revealed a trichobezoar with jejunal extension, causing transient jejunojejunal intussusception. Exploratory laparotomy confirmed the diagnosis of Rapunzel syndrome, and the trichobezoar was removed via gastrotomy. Following extraction, the intussusception resolved spontaneously. The patient was discharged on postoperative Day 7 after an uneventful recovery. Psychiatric evaluation later identified trichotillomania, and behavioural therapy was initiated, with no recurrence. The rare association between trichobezoar and jejunojejunal intussusception underscores the need for prompt diagnosis, definitive surgical management, and psychiatric assessment to minimize the risk of recurrence.

## Introduction

Rapunzel syndrome was first described in 1968 and is a rare form of trichobezoar where a hairball in the stomach extends through the pylorus into the small intestine [1]. The name comes from the Brothers Grimm fairy tale, referring to the long, tail-like extension into the bowel [2]. This condition mostly affects teenage girls and young women who have trichotillomania and trichophagia [3–5].

Trichotillomania is characterized by recurrent, irresistible urges to pull out one’s hair, while trichophagia involves compulsive hair ingestion. Although both conditions are linked, trichotillomania is more prevalent. Not all individuals with trichotillomania progress to trichophagia. Studies suggest that only a subset develops trichophagia, and this progression can lead to serious clinical consequences because human hair is indigestible. Once ingested, hair resists peristalsis and becomes trapped in gastric folds. Over time, accumulated hair compacts into a mass that may extend through the pylorus into the duodenum and jejunum [6]. Understanding the differing prevalence and risk factors helps clinicians assess patients who may be at higher risk of complications such as Rapunzel syndrome.

Trichobezoars are frequently asymptomatic initially and may be misdiagnosed as tumours. As the mass enlarges, patients can develop abdominal pain, bloating, early satiety, nausea, vomiting, weight loss, and occasionally a detectable abdominal mass. If left untreated, severe complications may arise, including gastrointestinal perforation, intra-abdominal infection, pancreatitis, appendicitis, and intussusception [7]. Intussusception is especially alarming due to the risk of compromised bowel perfusion, tissue necrosis, and subsequent perforation.

Surgical treatment continues to be the primary treatment for large trichobezoars and Rapunzel syndrome, especially when complications such as obstruction or intussusception are present [7]. Psychiatric assessment and behavioural management are essential to reduce the risk of recurrence.

## Case presentation

A 20-year-old woman presented with a 3-month history of progressively worsening abdominal pain, intermittent vomiting, early satiety, generalized weakness, and a self-detected abdominal mass. Frequent vomiting led to reduced oral intake and impaired daily functioning. She had no prior history of abdominal surgery, psychiatric illness, or similar gastrointestinal complaints.

### Clinical examination and laboratory findings

Physical examination revealed a firm, non-tender, mobile mass in the left upper quadrant of the abdomen. There were no signs of peritonitis. Laboratory investigations were unremarkable except for mild anaemia. The chronicity of symptoms, presence of a palpable mass, and recurrent vomiting suggested an underlying anatomical gastrointestinal pathology.

**Figure 1:**
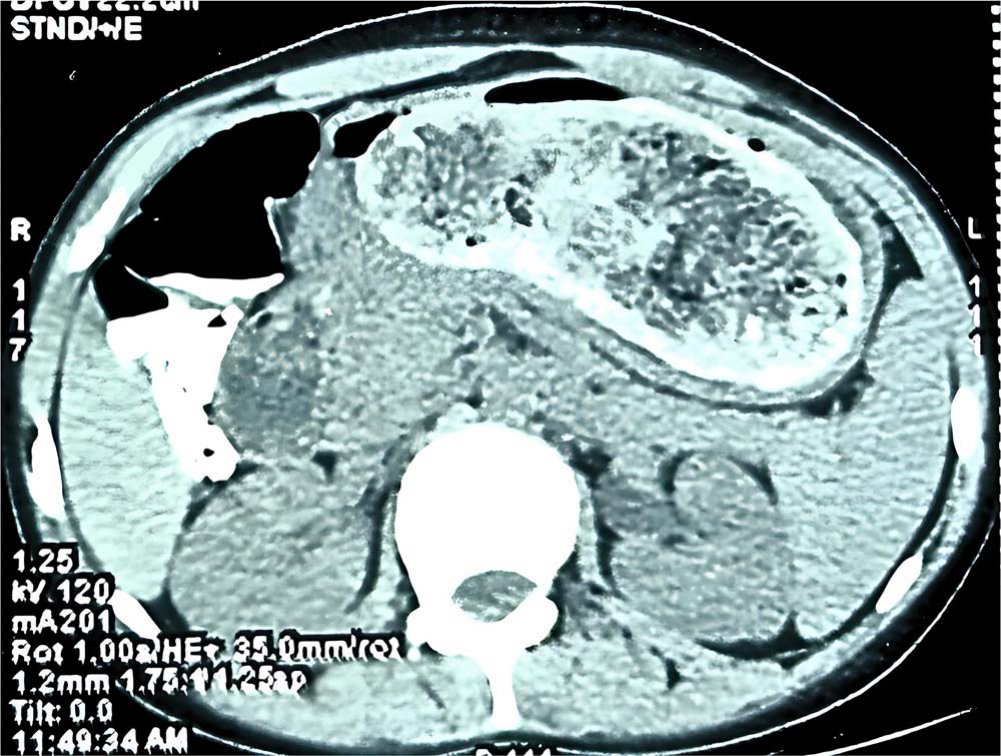
CT scan showing the bezoar within the stomach/intestine.

A contrast-enhanced CT scan of the abdomen (Fig. 1) showed a large, clearly outlined intraluminal mass measuring 50.6 × 114.6 × 82.1 mm, stretching from the stomach into the duodenum and jejunum. The mass had a mottled gas pattern, a feature typical of bezoars. The jejunum demonstrated a transient intussusception with proximal bowel dilatation and the classic ‘target sign’, supporting the diagnosis of Rapunzel syndrome with jejunojejunal intussusception.

This case is notable for the considerable size of the trichobezoar and its extensive extension throughout the gastrointestinal tract, reaching the distal jejunum and causing transient intussusception.

## Surgical management

**Figure 2:**
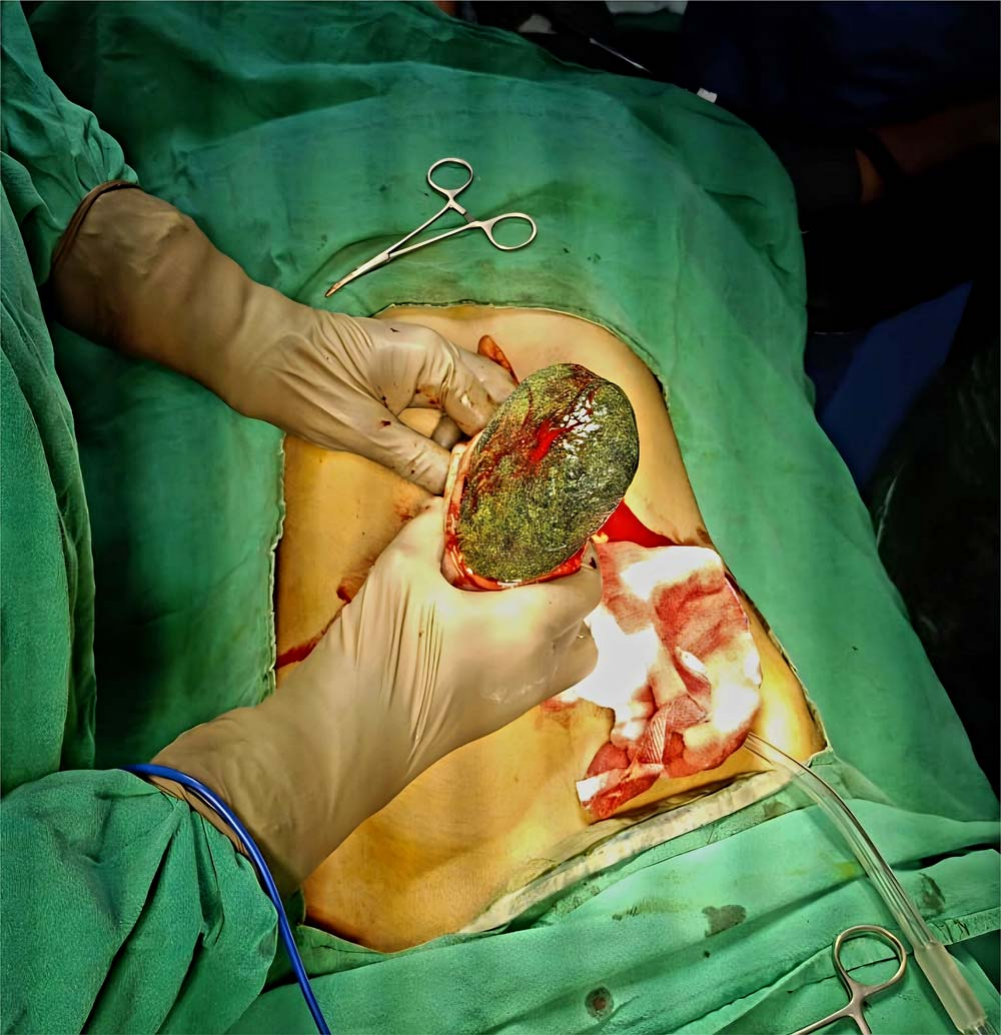
Intraoperative picture.

**Figure 3:**
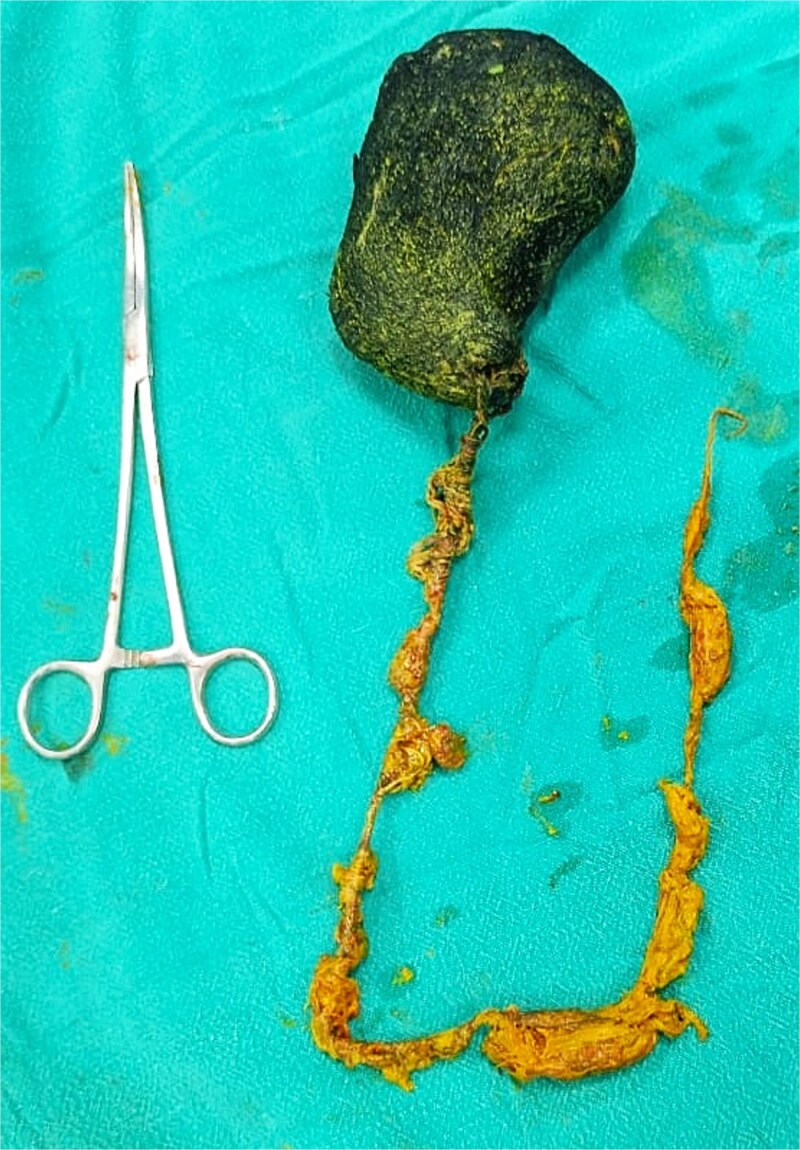
Hair ball with tail (Rapunzel syndrome). Note: Fewer than 10 cases of Rapunzel syndrome with jejunojejunal intussusception have been documented in the literature. The current case represents one of the youngest adult patients with this specific complication and demonstrates successful management with complete surgical removal and comprehensive psychiatric follow-up.

Due to the size of the mass and the presence of intussusception, exploratory laparotomy was performed via an upper midline incision (Fig. 2). Gastrotomy revealed a trichobezoar measuring ~12 × 8 cm with a 22 cm tail, occupying the stomach and extending into the jejunum. The entire mass was extracted through the gastrotomy (Fig. 3).

Following removal of the bezoar, the intussusception resolved spontaneously, indicating a mechanical lead-point aetiology rather than vascular compromise. No bowel resection was required. The gastric incision was closed in two layers.

### Postoperative course and follow-up

The postoperative course was uneventful. The patient was discharged on postoperative Day 7 and referred for psychiatric evaluation to assess for trichotillomania and implement measures to prevent recurrence.

At the 3-month follow-up, psychiatric assessment confirmed a diagnosis of trichotillomania and behavioural therapy was initiated. The patient remained asymptomatic with no evidence of recurrence, indicating good compliance with therapy. She will continue follow-up every 6 months for 2 years to monitor behavioural progress and recurrence.

## Discussion

Rapunzel syndrome is a rare type of trichobezoar in which a hairball in the stomach has a tail that extends into the small intestine [5, 8]. The condition develops when hair that cannot be digested collects in the stomach, becomes tangled, and forms a mass. As it grows, it can pass through the pylorus into the duodenum and jejunum [3, 5, 6]. This extension increases the risk of small bowel obstruction and may trigger intussusception.

In Rapunzel syndrome, intussusception occurs when the distal tail acts as a mechanical lead point, allowing peristalsis to draw one bowel segment into an adjacent segment, resulting in telescoping [4, 5, 8]. While intussusception is common in children, adult cases are uncommon and typically linked to pathological lead points such as tumours, polyps, Meckel’s diverticulum, or rarely bezoars. The association between Rapunzel syndrome and jejunojejunal intussusception is exceptionally rare, with fewer than 10 documented cases worldwide [2, 7] (Table 1). This rarity contributes to diagnostic difficulty and stresses the importance of considering bezoar-related pathology when radiology demonstrates characteristic intraluminal masses.

**Table 1:** Published cases of Rapunzel syndrome complicated by intussusception.

Author (year)	Ref	Age/Sex	Location of intussusception	Bezoar size	Extension	Psychiatric history	Surgical approach	Outcome
Vaughan *et al.* (1968)	[1]	17/F	Ileocolic	Not specified	Stomach to ileum	Not reported	Laparotomy, gastrotomy	Recovery
Schulte-Markwort *et al.* (2000)	[3]	16/F	Small bowel	15 cm gastric	Stomach to jejunum	Trichotillomania	Laparotomy, gastrotomy	Recovery, psychiatric follow-up
Schuler *et al*. (2023)	[5]	19/F	Not reported	15 cm with 30 cm tail	Stomach to jejunum	Trichotillomania	Open gastrotomy	Recovery, CBT initiated
Kouskos *et al*. (2024)	[8]	18/F	Not specified	Large gastric mass	Stomach to duodenum	Not reported	Laparotomy, gastrotomy	Uneventful recovery
Pokhrel *et al*. (2024)	[2]	4/F	Jejunojejunal	10 × 8 cm	Stomach to jejunum	Trichotillomania, pica	Exploratory laparotomy	Recovery, behavioral therapy
Agarwal *et al*. (2025)	[4]	22/F	Not complicated	12 × 10 cm	Stomach to jejunum	Trichotillomania suspected	Laparotomy, gastrotomy	Recovery, psychiatric referral
Current case (2025)	-	20/F	Jejunojejunal	12 × 8 cm; tail 22 cm	Stomach to jejunum	Trichotillomania (diagnosed post-op)	Laparotomy, gastrotomy	Recovery, behavioral therapy, no recurrence at 3 months

Abbreviations: F, female; CT, computed tomography; CECT, contrast-enhanced computed tomography; CBT, cognitive behavioural therapy; Ref, reference number.

Computed tomography (CT) is the best modality for diagnosis. CT typically shows a non-enhancing intraluminal mass with a mottled gas pattern due to trapped air within the hairball [2, 6]. It also demonstrates the extent of the bezoar, including the tail and associated complications such as obstruction or intussusception. In this case, CT showed both the trichobezoar and transient jejunojejunal intussusception, aiding surgical planning. The ‘target sign’ confirmed the intussusception, and proximal bowel dilatation indicated obstruction.

Management depends on the size, location, and extension of the bezoar. Small gastric trichobezoars may be attempted endoscopically, but this is often unsuccessful when the mass is large, densely compacted, or extends beyond the pylorus. Fragmentation may leave residual pieces that migrate distally and worsen obstruction. For Rapunzel syndrome, complete surgical removal of the trichobezoar and its tail remains the treatment of choice, particularly in the presence of complications [2, 4, 6, 8]. In this case, the bezoar was completely removed via gastrotomy and the intussusception resolved spontaneously after removal of the lead point. Early intervention likely prevented bowel ischemia and avoided the need for resection.

Prevention of recurrence is essential for long-term management. Psychiatric follow-up is important because the behaviours underlying the condition—trichotillomania and trichophagia—often persist without treatment. Recurrence is common if left untreated [7]. Current management focuses on cognitive behavioural therapy and habit-reversal training, with pharmacotherapy considered when indicated. In this case, structured behavioural therapy led to symptom-free follow-up at 3 months (Table 2).

**Table 2:** Clinical characteristics and management strategies in Rapunzel syndrome studies.

Study	Ref	Year	Sample size	Mean age (years)	Female (%)	Diagnostic modality	Most common symptoms	Endoscopic removal success rate	Surgical intervention rate	Recurrence rate	Psychiatric follow-up recommended
Vaughan *et al*.	[1]	1968	1	17	100	Clinical exam, imaging	Abdominal pain, mass	Not attempted	100%	Not reported	Not mentioned
Sehgal and Srivastava	[7]	2006	Review	Variable	90–95	CT, endoscopy	Pain, vomiting, mass	10%–30% for small bezoars	70%–90%	10%–20%	Strongly recommended
Snorrason *et al*.	[6]	2022	120 (review)	12.5	88	CT, ultrasound, endoscopy	Abdominal pain, nausea, mass	Limited success	85%	15%–25% without psychiatric care	Essential
Schuler *et al*.	[5]	2023	1	19	100	CT	Pain, vomiting, early satiety	Not attempted	100%	None at 6 months	Yes—CBT
Pokhrel *et al*.	[2]	2024	1	4	100	CT, ultrasound	Obstruction, vomiting, pain	Not attempted	100%	None at follow-up	Yes—behavioral therapy
Agarwal *et al*.	[4]	2025	1	22	100	CECT	Pain, mass, early satiety	Not attempted	100%	Not reported	Yes—recommended
Current study	-	2025	1	20	100	CECT	Pain, vomiting, early satiety, mass	Not attempted	100%	None at 3 months	Yes —biweekly sessions initiated

Abbreviations: F, female; CT, computed tomography; CECT, contrast-enhanced computed tomography; CBT, cognitive behavioural therapy; Ref, reference number.

## Conclusion

Rapunzel syndrome with jejunojejunal intussusception is an exceptionally rare condition in which a gastric trichobezoar extends into the small intestine. The tail of the trichobezoar acts as a lead point, precipitating intussusception and obstruction. Patients typically present with persistent abdominal pain, early satiety, recurrent vomiting, and a palpable mass. Early recognition is critical, particularly in young women, even in the absence of a known psychiatric diagnosis.

CT imaging plays a central role in diagnosis by demonstrating a non-enhancing intraluminal mass with a mottled gas pattern and tail-like extension. Complete surgical removal remains the definitive treatment, with bowel resection reserved for cases of ischemia or necrosis. Psychiatric assessment and behavioural therapy are equally important to reduce recurrence risk.

Continued documentation of rare cases and long-term follow-up will improve understanding of optimal diagnostic and management strategies. Clinicians should maintain a high index of suspicion for trichobezoar in similar presentations to ensure timely and comprehensive patient care.
